# Correction: Hood, M.A., *et al*. Synthetic Strategies in the Preparation of Polymer/Inorganic Hybrid Nanoparticles. *Materials* 2014, *7*, 4057–4087

**DOI:** 10.3390/ma7117583

**Published:** 2014-11-24

**Authors:** Materials Editorial Office

**Affiliations:** Klybeckstrasse 64, CH-4057 Basel, Switzerland; E-Mail: materials@mdpi.com

In [1], several sentences were repeated three times on pages 4062, 4063 and 4065. In addition, many references were incorrect. The errors were introduced by the editorial office during the editing process. We apologize for this mistake and any inconvenience this may have caused to authors and readers. The corrected manuscript is given below.

## Figures and Tables

**Figure 1.**
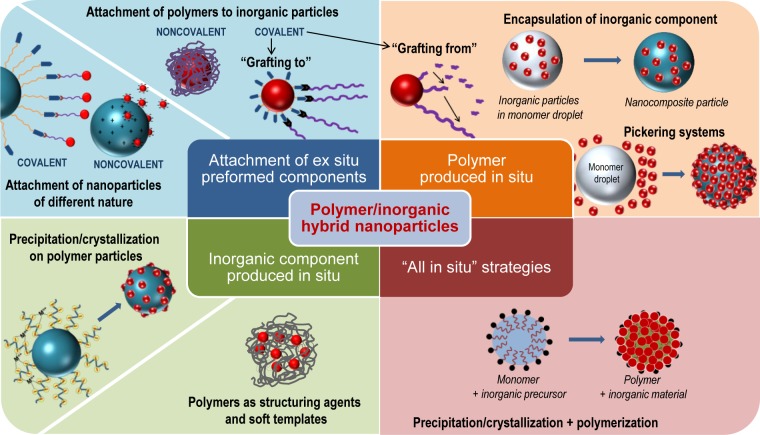
Different synthetic strategies in the formation of polymer/inorganic hybrid particles.

**Figure 2.**
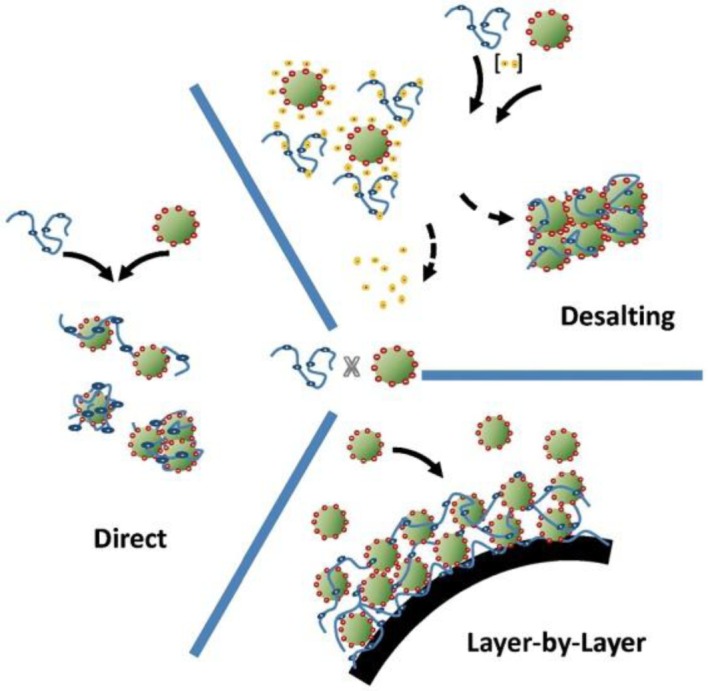
Summary of the three methods to form thin layers of physically attached polymeric chains to inorganic particles by taking advantage of charge. Reprinted with permission from [16]. Copyright 2012, Elsevier.

**Figure 3.**
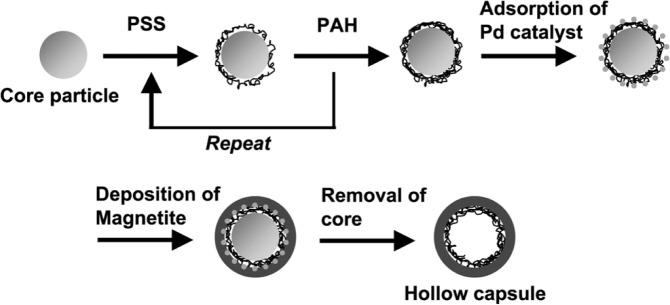
Formation of Fe_3_O_4_/Pd/polyelectrolyte hybrid capsules by layer-by-layer deposition. Reprinted with permission from from [29]. Copyright 2010, Elsevier.

**Figure 4.**
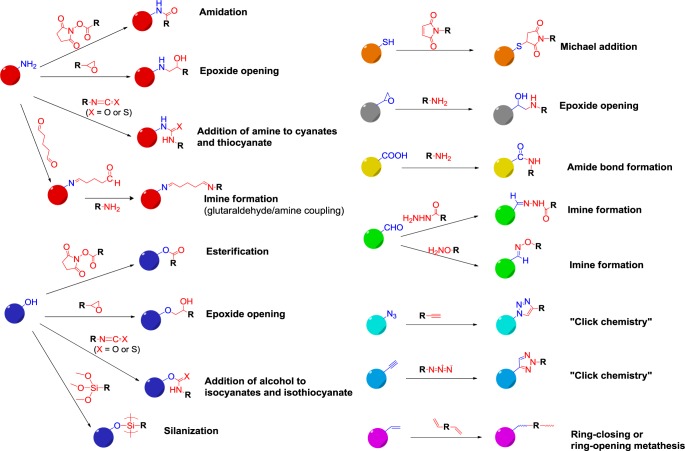
Main strategies for covalent post-functionalization of nanoparticles. Reproduced with permission from [20], partially based on Erathodiyil and Ying [37]. Copyright 2013, Eureka Science Ltd.

**Figure 5.**
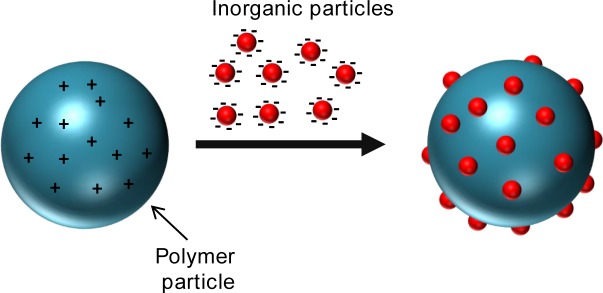
Heterocoagulation of oppositely charged colloids.

**Figure 6.**
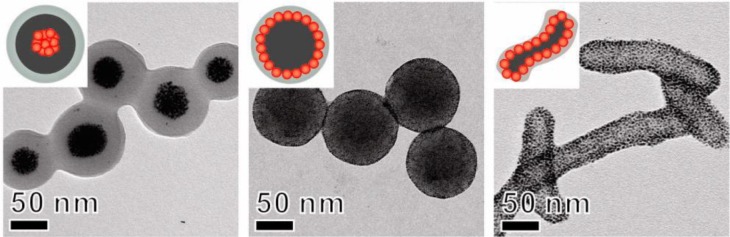
Using inorganic particle surface modification to control the placement of gold nanoparticles in a polysytrene-*block*-poly(acrylic acid) copolymer. Reprinted with permission from [48]. Copyright 2013, American Chemical Society.

**Figure 7.**
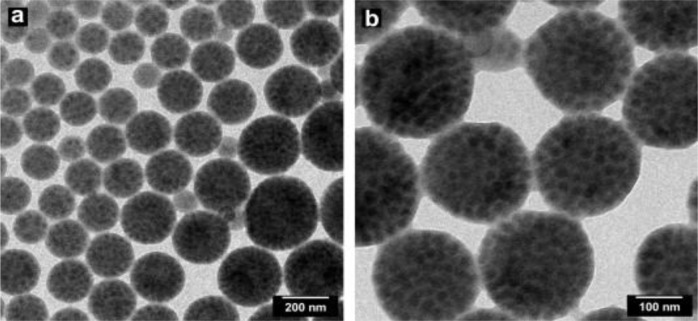
Morphology of hybrid particles with 40 wt% silica and good size distribution. Panels (**a**) and (**b**) show TEM images at different magnifications. Reprinted with permission from [57]. Copyright 2013, Elsevier.

**Figure 8.**
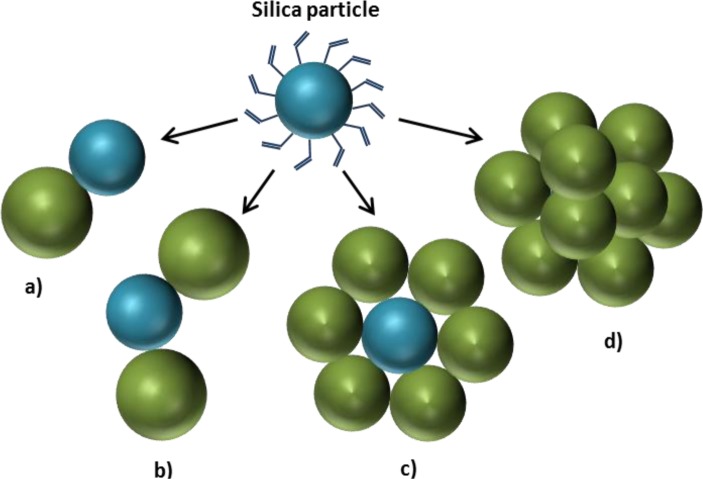
Morphology control over polystyrene growth on silica nanoparticles: (**a**) polystyrene/silica ratio equal to 1; (**b**) polystyrene/silica ratio equal to 2; (**c**) polystyrene/silica ratio equal to 6; (**d**) polystyrene in excess respect to silica. (Self-drawn scheme based on the strategy reported in [60,61].)

**Figure 9.**
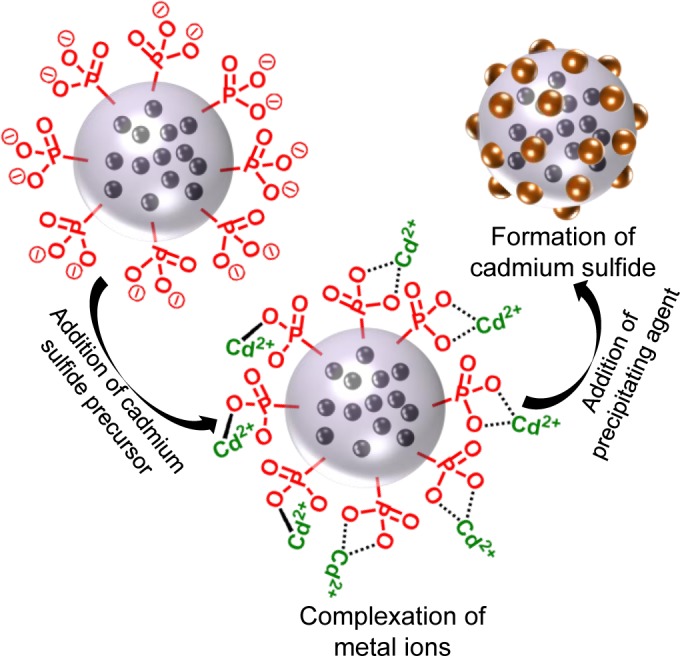
Mechanism of metal chalcogenide formation at the surface of phosphonate functionalized latex particles with magnetic iron oxide core. Reprinted with permission from [108]. Copyright 2013, American Chemical Society.

**Figure 10.**
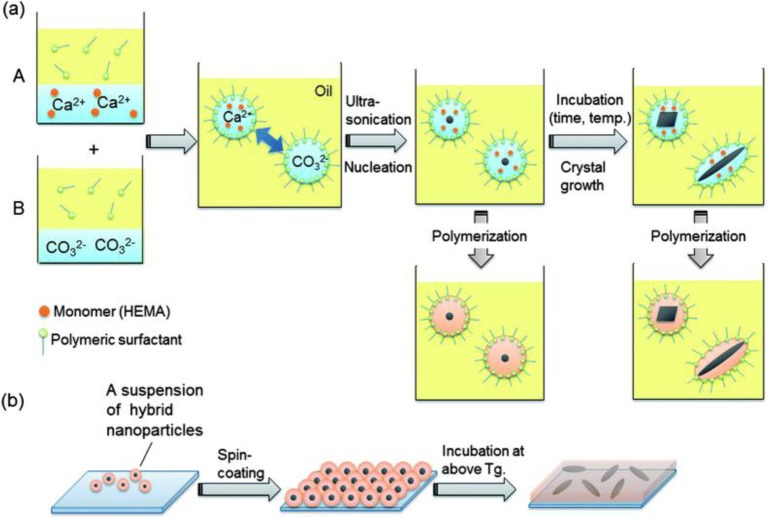
(**a**) Nanodroplets containing Ca^2+^/HEMA were added to nanodroplets containing CO_3_^2−^ and then mixed to start nucleation of CaCO_3_. Crystal growth was tunable by incubation with HEMA as a monomer and subsequent polymerization would give rise to nano-CaCO_3_-encapsulated hybrid nanoparticles with a variety of crystal shapes and structures; (**b**) preparation of a hybrid nanofilm via the spin-coating of a suspension of the hybrid nanoparticles. Reproduced with permission from [125]. Copyright 2012, the Royal Society of Chemistry.

**Figure 11.**
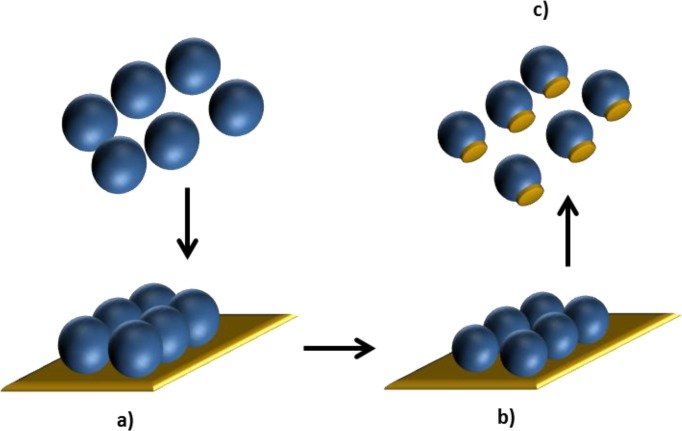
Hybrid polystyrene/gold nanoparticles prepared by: (**a**) adhesion of the particles on Au layer; (**b**) size reduction by plasma *etc*hing; (**c**) release from substrate. Self-drawn scheme based on the strategy reported in [128].

**Table 1.** Overview publication on specific issues related with the synthesis and applications of hybrid nanoparticles.

Year	Authors	Reference	Main aspect highlighted
2001	F. Caruso	[2]	Functionalization of particle surfaces (mainly layer-by-layer)

2004	F. Caruso (ed.)	[3]	Colloidal particles (book)
	Shi *et al*.	[4]	Layer-by-layer technique for nanostructured materials

2007	Ballauff and Lu	[5]	Thermosensitive core–shell microgel particles (also hybrid particles)
	Lattuada and Hatton	[6]	Janus magnetic nanoparticles

2009	Karg and Hellweg	[7]	Poly(NIPAM) microgels and nanoparticle microgel hybrids
	Landfester	[8]	Polymer and hybrid nanoparticles by miniemulsion polymerization

2010	Agrawal *et al*.	[9]	Colloid-based composite particles
	Landfester and Weiss	[10]	Encapsulation in nanoparticles by miniemulsion polymerization
	Sperling and Parak	[11]	Functionalization and bioconjugation of inorganic nanoparticles
	Than and Green	[12]	Functionalization of nanoparticles for biomedical applications

2011	Hu *et al*.	[13]	Organic–inorganic nanocomposites by miniemulsion polymerization
	Musyanovych and Landfester	[14]	Core–shell particles
	Neoh and Kang	[15]	Polymer-functionalized inorganic nanoparticles for biomedical applications

2012	Chapel and Berret	[16]	Electrostatic assembly of nanoparticles and polyelectrolytes
	Dong *et al*.	[17]	Soft vesicles as templates for inorganic materials
	Muñoz-Espí *et al*.	[18]	Miniemulsion for inorganic synthesis
	Sailor and Park	[19]	Hybrid nanoparticles for detection and treatment of cancer

2013	Froimowicz *et al*.	[20]	General overview of surface-functionalized nanoparticles
	He *et al*.	[21]	Asymmetric metal(oxide) hybrid nanoparticles
	Muñoz-Espí *et al*.	[22]	Use of colloidal systems for crystallization

2014	Rangelov and Pispas	[23]	Polymer and polymer-hybrid nanoparticles (book)

**Table 2.** Representative works on the formation of polymer/inorganic hybrid particles by *in situ* precipitation of inorganic materials on the surface of colloid polymer particles.

Inorganic material	Polymer support ^[a]^	Precipitation solvent	Approximate size of hybrid particles/nm	Reference
Ag	poly(S/NIPAM)	water	~120 (core)	[98]

Au	poly(S/AEMH)	water	80–90 (core)	[99,100]

Au, Ag	PS+PEI	water	>400	[101]
	PS	ethanol/acetone	~710	[96]

Au–Pt	poly(S/NIPAM)	water	200 (core)	[102]
	poly(S/AEMH)		80–90 (core)	[103]

Ca_5_(PO_4_)_3_(OH)	poly(S/AA)	water	>360	[95]
	poly(S/AA)		100–350	[104]
	poly(S/AAEMA)		>640	[105]
	poly(S/VPA) or poly(S/VBPA)		~200–300	[106]
	poly(S/R–PO_3_H_2_)		180–270	[107]
	poly(S/R–PO_3_H_2_)		100–250	[107]

CdS	poly(S/R–PO_3_H_2_)	water	140–180	[108]

CeO_2_	poly(S/R–PO_3_H_2_) or poly(S/R–PO_4_H_2_)	water	140–200	[109]

Co(OH)_3_	PS	water	>600 nm	[110]

Fe_2_O_3_	poly(S/R–PO_3_H_2_) or poly(S/R–PO_4_H_2_)	water, 2-propanol	140–200	[109]

Fe_3_O_4_	Sulfate-stabilized PS	water/ethylene glycol	~220	[97]
	Poly(S/RPO_3_H_2_) or poly(S/RPO_4_H_2_)	water	140–200	[109]

Fe(OH)_3_	PS	water	>600 nm	[110]

In(OH)_3_	Poly(S/AAEMA)	2-propanol	700–750	[111]

LiNbO_3_	PS/PDADMAC/PSS	ethanol, 2-propanol	640	[112]

Pd	P(S/MPTAC)	water	<100 (core)	[113]

Pt	P(S/MPTAC)	water	<100 core	[114]
	P(S/AEMH)		100 core	[103]

Ta_2_O_5_	poly(S/AAEMA)	ethanol	550–850	[115]

TiO_2_	PS/PDADMAC/PSS	water	640	[116]
	PS (sulfonated)	ethanol/water	100–500	[117]
	poly(S/AAEMA)	ethanol, 70 °c	~600–700	[118]
	PS (SDS-stabilized + –COOH groups)	ethanol/H_2_O	120	[119]
	Poly(S/SS)	ethanol	~160	[120]

Y(OH)CO_3_	PS (cationic-anionic)	water	170–360	[93]

ZnO	Poly(S/AAEMA)	2-propanol	300–700	[121]
	poly(S/R–PO_3_H_2_) or poly(S/R–PO_4_H_2_)	methanol, 2-propanol or ethanol	140–200	[109]

ZnO/TiO_2_	poly(S/AAEMA)	2-propanol/ethanol	~600–700	[122]

Zr_2_O_2_(OH)_2_CO_3_, Zr_2_(OH)_6_SO_4_	PS (cationic/anionic)	water (+formamide)	190–260	[94]

ZnS	P(S/AAEMA) and PS/PGEMA)	water	~350–600	[123]

[a] AAEMA: acetoacetoxyethylmethacrylate; AEMH: 2-aminoethyl methacrylate hydrochloride; MPTAC: (2-methylpropenoyloxyethyl) trimethylammonium chloride; NIPAM: *N*-isopropylacrylamide; PDADMAC: poly(diallyl dimethyl ammonium chloride); PEI: poly(ethylene imine); PGEMA: poly(ethyleneglycol) methacrylate; PS: polystyrene; PSS: poly(styrene sulfonate); VBPA: vinylbenzylphosphonic acid; VPA: vinylphosphonic acid; S: styrene; SS: styrene sodium sulfonate.
