# Supplementary material for: Correction: Hood, M.A., et al. Synthetic Strategies in the Preparation of Polymer/Inorganic Hybrid Nanoparticles. Materials 2014, 7, 4057–4087
Source: Materials (Basel). 2014 Nov 24;7(11):7583–614. doi: 10.3390/ma7117583 (PMC5512675; doi:10.3390/ma7117583)
Supplement: Related texts

# Review article: Synthetic Strategies in the Preparation of Polymer/Inorganic Hybrid Nanoparticles

**DOI:** 10.3390/ma7054057

**Published:** 2014-05-22

**Authors:** Matthew A. Hood, Margherita Mari, Rafael Muñoz-Espí

**Affiliations:** Max Planck Institute for Polymer Research, Ackermannweg 10, 55118 Mainz, Germany; E-Mails: hood@mpip-mainz.mpg.de (M.A.H.); mari@mpip-mainz.mpg.de (M.M.)

**Keywords:** organic–inorganic, hybrid, nanoparticle, nanocomposite, colloid, precipitation

## Abstract

This article reviews the recent advances and challenges in the preparation of polymer/inorganic hybrid nanoparticles. We mainly focus on synthetic strategies, basing our classification on whether the inorganic and the polymer components have been formed *in situ* or *ex situ*, of the hybrid material. Accordingly, four types of strategies are identified and described, referring to recent examples: (i) *ex situ* formation of the components and subsequent attachment or integration, either by covalent or noncovalent bonding; (ii) *in situ* polymerization in the presence of *ex situ* formed inorganic nanoparticles; (iii) *in situ* precipitation of the inorganic components *on* or *in* polymer structures; and (iv) strategies in which both polymer and inorganic component are simultaneously formed *in situ*.

## 1. Introduction

Although the term “organic/inorganic hybrid nanomaterial” has become incredibly popular in the last few decades, the combination of organic and inorganic matter at the nanoscopic scale is not new. Indeed, nature has been fabricating hybrid materials since the origins of life. Bone, nacre, and corals are just a few representative examples of how the interaction of biogenic molecules with inorganic components is able to deliver hybrid biomaterials with a sophistication that is very difficult to replicate by humans. Herein, we aim to review recent synthetic advances in a specific area of organic–inorganic hybrid materials: the preparation of polymer/inorganic hybrid nanoparticles. We will refer exclusively to hybrid particles—and very specifically to those in the submicron and nanoscopic range—containing polymers as the organic component, leaving aside hybrid nanoparticles in which the organic component is made of smaller molecules.

Polymer/inorganic hybrid nanoparticles find their application in very diverse areas, including coatings, catalysis, optics, optoelectronics, and biomedical applications. In very general terms, the polymer component typically has structural functions and may tune the mechanical features and processability of the final materials, whereas the inorganic component can introduce specific functionalities (catalytic activity, luminescence, magnetism, *etc*.) and/or reinforce the mechanical and thermal properties of the polymer. The final properties of the hybrid nanoparticles are very often not a simple addition of the properties of the independent component, but a unique result from synergetic effects.

We have structured our article on the basis of the synthetic strategy used in producing hybrid nanoparticles, being aware that any classification has a certain degree of arbitrarity and subjectivity. Our criterion has been whether the inorganic and the polymer components are formed *in situ* or *ex situ* of the hybrid nanoparticles. Some authors consider “hybrid materials” only those in which the components are formed *in situ* (e.g., the inorganic component is formed *in situ* by sol–gel methods), while the combination of *ex situ* formed materials are referred to as “composite materials”. This classification is certainly legitimate and is based on the definition of “hybrid material” given in the *IUPAC Gold Book* [1]: “Material composed of an intimate mixture of inorganic components, organic components, or both types of component. Note: The components usually interpenetrate on scales of less than 1 μm.” However, in the case of nanoparticles, the interpenetration occurs always “on scales of less than 1 μm” and the difference between “nanocomposite particle” and “hybrid nanoparticle” may be relatively diffuse. Therefore, we have decided to use “hybrid nanoparticle” in a general way, even though some works reported here could also be, strictly speaking, considered as a “nanocomposite”.

We distinguish four groups of possible strategies in the formation of polymer/inorganic hybrid nanoparticles:

(1) Strategies in which the formation of both polymer and inorganic components takes place *ex situ*, and the hybrid nanoparticle is formed by their combination. Two cases can be differentiated: (i) those in which polymer chains are attached to inorganic particles; and (ii) those in which polymer nanoparticles and inorganic nanoparticles are combined together. In both cases, either with polymer chains or with particles, the attachment of the components can be *noncovalent* or *covalent*. (see Section 3)
(2) Strategies in which the inorganic component (typically inorganic nanoparticles) is formed *ex situ* and, afterwards, the polymerization of the organic component takes places in its presence. ( see Section 4)
(3) Strategies in which the polymer or the polymer particles are formed *ex situ* and the precipitation/crystallization of the inorganic component takes place *in situ*. (see Section 5)
(4) Strategies in which both the polymer and the inorganic components are simultaneously formed *in situ*, during hybrid nanoparticle formation. (see Section 6)

These synthetic pathways, schematically depicted in Figure 1, are described in detail within the article. At the end, we will also briefly refer to the assembly of nanoparticles, which has become a trending topic.

## 2. Literature on Organic/Inorganic Hybrid Particles and Related Topics

The topic of hybrid nanoparticles is extensive and it would be impossible to exhaustively discuss all works to date. Our objective is, therefore, more modest: we aim to highlight examples that, under our point of view, are representative of the different preparation strategies of polymer/inorganic hybrid nanoparticles. Several books, feature articles, and reviews on specific issues related with the synthesis and applications of hybrid nanoparticles have been published in recent years. To prevent repetition, we address the reader to those references, where appropriate. Some of the most recent overview publications in the topic are listed in Table 1. Many of the works of this table do not refer exclusively to hybrid nanoparticles, but contain relevant sections related to the topic. Most of these publications either have a general scope or are very specific (e.g., on concrete applications), and a comprehensive overview focusing exclusively on recent advances the preparation of polymer/inorganic hybrid nanoparticle has been missing. The present review tries to fill this gap, serving as an introduction for undergraduate and graduate students.

## 3. Preparation of Hybrid Polymer/Inorganic Nanoparticles from *Ex Situ* Produced Components

### 3.1. Attachment of Polymer Chains to Inorganic Nanoparticles

Organic molecules have been used extensively to modify inorganic nanoparticles, whether as small ligands to tailor the surface properties of inorganic nanoparticles or as longer polymeric chains that aid in the formation of new polymer/inorganic hybrid materials. The attachment of polymers to inorganic nanoparticles can either by physical (noncovalent) or by covalent means, and the resulting hybrid material has unique properties depending on the synthetic route.

#### 3.1.1. Noncovalent Attachment

Polymeric coatings on inorganic nanoparticles can be prepared by taking advantage of the electrostatic interactions that occur between a charged polymer (polyelectrolytes and ionomers) and the charged surface of an inorganic particle. A good review on deposition of polymer chains onto nanoparticles has been written by Chapel and Berret [16]. Figure 2 shows the three main methods highlighted by these authors in using electrostatic interactions of polymers and nanoparticles in forming coatings and, thus, hybrid nanoparticles: (i) direct addition; (ii) desalting; and (iii) layer-by-layer. The direct addition consists of the mixing of both components together to form aggregates of the two oppositely charged species. In a desalting process, both the inorganic particles and the polymers are dispersed within a medium and are stabilized from interacting by the presence of salt ions. The removal of the salt allows for a more ordered aggregation to begin, because the desalting rate can be controlled. Finally, the layer-by-layer (LbL) deposition allows the formation of highly controlled coatings, which can yield attractive hierarchical morphologies, but through a quite time-consuming process.

The LbL technique implies the addition of one charged species into a concentrated suspension of the oppositely charged species. The process is repeated two or more times, thus forming a controlled number of oppositely charged layers on the particle surface. Weakly attached species are washed away after each layer deposition, which is time consuming. In recent years, automation of this process has led to an increased processing rate [24,25].

LbL techniques in the formation of functional hybrid particles for use in drug delivery applications have been recently attracting a lot of attention. Tong *et al*. [26] have reviewed the use of LbL deposition in nanoparticles for biomedical applications. The versatility of the method has enabled the design and preparation of shell structures with a variety of compositions and functionalities on the surface of multiple inorganic particles, including ZnO nanorod-based hybrid nanomaterials prepared at room temperature with cores made of ZnO/CeO_2_, ZnO/CdS, and ZnO/Ag [27].

The formation of hybrid nanocapsules is also possible by applying the LbL approach. Hollow silica and silica/polymer spheres were formed by deposition of silica nanoparticles and polymer onto colloidal templates. The wall thickness of the capsules can be controlled by varying the number of nanoparticle and polymer deposition cycles. By changing the morphology and size of the colloid template, the capsule structure can also be varied [28]. Nakamura *et al*. [29] prepared Fe_3_O_4_/Pd/polyelectrolyte hybrid capsules by this technique (Figure 3). LbL assembly of polyelectrolytes onto melamine/formaldehyde particles was followed by the adsorption of palladium catalysts onto the poly(allylamine hydrochloride)-terminated surface. Afterwards, Fe_3_O_4_ nanoparticles were deposited on the top of the structure. Fe_3_O_4_/polyelectrolyte hybrid capsules can be particularly interesting for medical applications.

The formation of polymeric layers onto particles is a well-established technique now and it is expected that its use will continue to be explored with great interest, because the morphology and the thickness of the layers can be easily controlled by using commercially available materials without need of any demanding chemical reaction. LbL deposition may also be used to deposit inorganic materials formed *in situ* onto preformed polymer particles, which will be discussed in Section 5.2.

#### 3.1.2. Covalent Attachment

Single layer coatings of covalently attached polymeric chains to particles surfaces fall under the category of the so-called ‘polymer brushes’, which have been well studied and reviewed [30,31,32]. Polymer brushes have three regimes: Gaussian random coils, a mushroom regime, and the polymer brush structure, which are dependent on the increasing grafting density of the polymer coating to the inorganic particle [33]. The polymeric chains may be attached in two ways: either by “grafting to” or by “grafting from” approaches, which involve coupling techniques and surface-initialized polymerization, respectively. “Grafting from” methods have commonly focused on living polymerization or atom transfer radical polymerization, of which the works and reviews of Matyjaszewski’s group are representative [34–36]. Although “grafting to” and “grafting from” methods are typically explained together, as they relate both to polymer brushes, under our classification “grafting from” strategies belong to the *in situ* polymerization strategies and will be discussed in that section.

Coupling techniques—or “grafting to”—methods often require the addition of functional groups to the surface of inorganic nanoparticles, allowing then for conventional chemical reactions to graft polymers to the modified inorganic particles. In a recent publication, Froimowicz *et al*. [20] have reviewed post-functionalization strategies of existing nanoparticles. Figure 4 presents an overview of the most common reactions used for coupling. 

Among the different possibilities, “click chemistry” is useful in attaching chains to the surface of particles, as it can be carried out with high efficiency under relatively mild conditions [38]. A representative example of the ease of click chemistry as a means of attaining polymer brushes on inorganic particles was shown by Tchoul *et al*. [39], who used titania nanoparticles as a core with polystyrene grafted onto the surface. The titania surface was prepared by phosphonate coupling and subsequent “click chemistry” to attach the polystyrene chains to the surface of the modified particles. Additionally, “click chemistry” based on thiol-ene reactions have been used to modify nanocellulose crystals for compatiblizing into the matrix of composites materials [40]. More recently, “click reactions” along with *in situ* polymerization techniques, such as controlled radical polymerization, have been used together to form highly tailorable materials [41].

### 3.2. Hierarchical Hybrid Nanoparticles by Combination of Ex-Situ Formed Nanoparticles

In this section, we present the preparation of hybrid particles by interaction of preformed inorganic and polymer nanoparticles. This synthetic approach has only been lightly explored during the last years, but is gaining interest, because it allows the formation of nanomaterials with complex shapes and great morphological control. Like in the previous section, the various methods can be classified into two groups, depending on whether they involve *noncovalent* or *covalent bonding*. In the first case, the surface of both types of particles has to be modified with oppositely charged groups in order to have electrostatic interactions that lead to a homogeneous distribution of the inorganic nanoparticles on the polymer surface. In the second case, the nanoparticles are functionalized with complementary groups so that are able to react and bind.

#### 3.2.1. Noncovalent Attachment

An additional way to achieve the interaction between two materials of completely different natures is to modify their surfaces and then use the so-called “heterocoagulation” technique, schematically depicted in Figure 5. Heterocoagulation implies the interaction and aggregation of particles of different nature to form a hybrid product. Wagner *et al*. [42] reported raspberry-like particles obtained by heterocoagulation of binary mixtures of oppositely charged colloids. The authors used an organic core of cross-linked polystyrene particles functionalized at the surface with amino groups and three types of inorganic particles, namely anionic silica, gold precipitated in the presence of trisodium citrate dihydrate, and maghemite precipitated in the presence of poly(acrylic acid) and diethylene glycol. The process is simple: the polystyrene particles were added dropwise to a suspension of inorganic nanoparticles. The uniform distribution of inorganic particles on the surface of polystyrene spheres is explained by having the adsorbed inorganic nanoparticles located within the electrostatic double layer of the large polymeric particles, acting as multivalent counter-ions.

The same method was applied by Kanahara *et al*. [43] with citrate-coated gold nanoparticles on amino-terminated polystyrene and amino-terminated 1,2-polybutadiene (PB-NH_2_). Interestingly, while polystyrene-based hybrids show the inorganic functionalization clearly on the surface, with addition of PB-NH_2_ it is possible to control the penetration depth, which increases with low molecular weight of PH-NH_2_ and smaller diameter of the Au nanoparticles.

An alternative method to obtain hybrid particles was shown by Jiang *et al*. [44]. In their approach, silica nanoparticles were half-embedded in wax droplets. In an emulsion of molten wax and water, untreated hydrophilic fused silica particles adsorbed to the oil–water interface at an elevated temperature at which the wax was molten. After the particles had been fully adsorbed, the temperature was lowered to solidify the wax phase and embed the particles. This template can be useful for further partial functionalization of the silica particles to obtain so-called Janus morphologies (*i.e.*, asymmetric particles with two distinct parts). The use of a solidified oil phase offers the advantage of freezing the particles into fixed positions during the chemical modification step, avoiding the possibility that adsorbed particles wobble or rotate at the liquid–liquid interface.

#### 3.2.2. Covalent Attachment

Analogous to Section 3.1.2, the attachment between polymeric and inorganic nanoparticles can also be achieved by covalent bonding, which is more stable than electrostatic interactions. Figure 4 can also be applied here. However, the examples reported in literature are not as numerous as in the case of covalent attachment of polymer chains to inorganic nanoparticles, which is popular in bioconjugation strategies in biology [11]. An elegant example was reported by Agrawal *et al*. [45], who covalently attached amino-functionalized CdTe nanocrystals of about 3.2 nm to carboxylic-terminated poly(*N*-isopropyl acrylamide) (PNIPAM) microgel nanoparticles by amide coupling with a carbodiimide. In another example, alkyne–azide “click chemistry” was used to couple azide-modified gold nanoparticles to acetylene-functionalized polymer nanoparticles [46]. The authors demonstrated that the attachment of the gold particles was only successful when the coupling reaction was carried out in the presence of a copper, a catalyst for the aklyne-azide reaction.

### 3.3. Entrapment of Inorganic Nanoparticles by Nanostructures Formed by Polymers

More complex organic superstructures can be used to form hybrid systems containing nanoparticles in a specific region. The use of dendrimers gives the polymer matrix a degree of control that is not possible with typical *in situ* polymerization or cross-linking techniques for encapsulation. Surface-modified quantum dots were shown to be placed in a specific region of a matrix composed of amphiphilic dendrimers. Interesting vesicle–shell or core–shell structures occurred due to phase separation of the dendrimers and the placement of the quantum dots depended on whether the quantum dots were modified with hydrophobic or hydrophilic components [47].

The modification of the inorganic particle surface is useful in controlling the location of inorganic particles in block-copolymer particles, as seen in Figure 6. By either using gold nanoparticles as-received or by modifying the surface with a hydrophobic ligand (dodecanethiol), it was possible to selectively place the nanoparticles into either polystyrene or poly(acrylic acid) blocks of a polysytrene-*block*-poly(acrylic acid) copolymer particle [48]. In addition to selecting appropriate surface modifications of inorganic particles, the tailoring of the compatibility between the inorganic component and the polymer matrix can be achieved by tuning the polymer composition and formulation. Hybrid particles were formed by encapsulating gold nanoparticles in a polystyrene particle. Addition of the block copolymer polystyrene-*block*-poly(4-vinylpyridine) and the small molecule 3-*n*-pentadecylphenol aided in increasing the gold loading in the hybrid nanoparticles up to values as high as 84 vol% [49].

Hybrid nanoparticles are often used for biomedical applications, such as drug delivery. Peptide-based hydrogel particles were cross-linked to encapsulate Au nanoparticles [50]. Gold can act here as a contrast agent for medical testing, while the biocompatible hydrogel network has a significant potential for additional bioactive properties. The release of drugs as a result of the swellability of the peptides was also demonstrated for such particles.

## 4. Hybrid Particle Formation by Polymerization in the Presence of *Ex Situ* Formed Inorganic Nanoparticles

Key in the formation of polymer/inorganic hybrids is the tailoring between the thermodynamics of the inorganic particles and the polymer matrix, so that the inorganic component is well wetted by the polymer chains and prevented from aggregating. By finely tuning the thermodynamic properties of the inorganic particles to match that of the polymer matrix, via particle size and surface functionality, it is possible to achieve select control over particle location within homopolymers, block copolymers, *etc*. [51]. Small changes in the compatibilizing agent can control the location of inorganic particles, either within the polymer particle matrix, at the interface of the polymer, or even both. The “grafting from” methods, shortly referred to in Section 3.2.2. are one of the possibilities for the *in situ* formation of polymer chains on the surface of inorganic nanoparticles. In the following section, however, we will rather focus on the formation of polymer particles in the presence of inorganic nanoparticles, mainly by applying heterophase methods.

### 4.1. Encapsulation of Inorganic Nanoparticles

A relatively conventional method in the production of hybrid polymer/inorganic particles has been the entrapping or incorporation of preformed inorganic nanoparticles during the polymerization of a polymer network. Such entrapping or encapsulation allows for inorganic particles to be either physically trapped within the matrix or covalently bound to the polymers. During many of these processes, the polymer chains grow as a network around the inorganic particles, making them structurally different from inorganic particles with polymer brushes, described earlier. When inorganic particles act as a cross-linking agent, they may significantly enhance the mechanical properties of the hybrid system [52]. It should be pointed out that for certain properties, the addition of the polymer layers may have an effect on the intrinsic properties of the functional inorganic particles. For example, the magnetic properties of nickel/polyacrylamide prepared by inverse miniemulsion technique were slightly diminished from those of pure nickel nanoparticles, mostly due to the presence of the polymer [53].

Unlike brush formation, incorporation of particles by emulsion processes is often a facile method and may be very useful in attaining hybrid particles for many commercial applications. Emulsions are formed by the stabilization of two immiscible liquids by means of an amphiphilic surfactant molecule. Typically, a considerable amount of surfactant molecules, which possess a lyophilic part and a hydrophilic part, are necessary to stabilize the dispersed phase from its thermodynamically incompatible continuous phase. When compared to conventional emulsions (also referred to as macroemulsions) and microemulsions, the surfactant concentration in miniemulsions is significantly lower [8]. If there is too little surfactant, coalescence will change the size of the droplets, but if the concentration is too high, then micelles can form and lead to micellar nucleation. The surfactant, although necessary, is often undesirable in the final hybrid material, as it may affect the final properties. Thus, when incorporating inorganic particles by emulsion and miniemulsion processes, it is important to take into account the surfactant concentration [54,55].

It has been shown that during the formation of hybrid particles by miniemulsion polymerization, a chemical modification of the inorganic particles is preferable to physical modification in order to produce stable, well dispersed inorganic particles within the polymer matrix [55]. As mentioned above, the type and extent of modification of the inorganic particles can also have a drastic effect on the location of the inorganic particles within the polymer matrix. Hydrophobically modified silica particles were encapsulated by polymerization of methyl methacrylate [55–57]. Bourgeat-Lami *et al*. [56] showed that it is possible to gain some control over the location of the silica particles within a poly(methyl methacrylate) nanoparticle by changing the surface modification with γ-methacryloxypropyltrimethoxysilane (MPS) and by introducing *n*-butyl acrylate as a comonomer to the methyl methacrylate droplet at varying concentrations. It was seen, however, that aggregation occurred within pure *n*-butyl acrylate monomer droplets or when the silica particles preferred to go to the oil–water interface, probably due to an incomplete hydrophobization of the inorganic surface. It is, therefore, important to characterize the surface properties and the extent of particle modification to control the compatibilization of the inorganic particle surface with the polymer matrix.

Prior to polymerization, it is often a challenge to break up monomer droplets containing inorganic nanoparticles. The increased viscosity and abrasiveness of the droplets has to be dealt with in order to avoid damage to the shear force generating equipment. Agglomerates of the droplets hinder deformation and break-up, which leads to large, nonspherical droplets. A process to produce small droplets with high inorganic particle concentrations without producing abrasive particles was developed by adjusting the surfactant concentration. Secondary nucleation was avoided and a final product with a high homogeneity could be achieved (Figure 7) [57].

Miniemulsion polymerization in the presence of hydrophobized inorganic nanoparticles has also been used in the preparation of magnetic nanoparticles. By using this technique, Ramírez and Landfester [58] reported the encapsulation of oleic-capped magnetite in polystyrene particles. Similar magnetic nanoparticles made of poly(l-lactic acid) have also been reported by Urban *et al*. [59], but in this case the polymer had been formed *ex-situ* and the particles were produced by solvent evaporation in miniemulsion.

### 4.2. Inorganic Particles as Seeds for Complex Hybrid Morphologies

An interesting example was reported by Ravaine *et al*. [60,61], who were able to obtain different numbers of polystyrene nodules associated to one silica particle, accomplishing a good control of morphology. Silica nanoparticles were prepared with the Stöber process and coated with polymerizable groups from which the growth of polystyrene grains started. The ability to have a controlled number of polymeric particles on the silica surface is the most significant feature of this method. The number of polymer nodules was constant, so that different morphologies can be obtained by varying the amount of silica (Figure 8). When the ratio between silica particles and the polymer nodules was 1, a snowman-like morphology was obtained, with only one polystyrene nodule growing per silica particle. By increasing the ratio to 2, the number of nodules per silica particles was also two. At a ratio of 6, a daisy-like shape was reached. An excess of polystyrene particles gave raspberry-like morphologies.

By using a similar strategy, polymer/laponite composite latexes have been prepared [62]. Laponite is a synthetic clay in the form of platelets that in water forms a homogeneous gel. Each laponite platelet, functionalized with an initiator, acted as a seed for the emulsion polymerization of poly(styrene-*co*-butyl acrylate). The final hybrid structures presented a layer of clay with a honeycomb-like network covering the nanoparticles. The superficial distribution of the inorganic fraction was reported to enhance the thermal stability, slowing down the transmission of heat and facilitating film formation.

Quiang *et al*. [63] applied the same synthetic concept to attach, in a one-step process, polystyrene nanoparticles produced by miniemulsion polymerization to silica nanoparticles functionalized with *n*-octadecyltrimethoxysilane. The hybrid nanoparticles showed a mushroom-cap-like shape for the polymer, resulting in a peculiar Janus morphology. Another interesting example of mushroom-like morphology was reported by Feyen *et al*. [64], who used Fe_3_O_4_ nanoparticles for the growth of poly(styrene-*co*-divinylbenzene). Polymeric spheres with one magnetic nanoparticle located on the surface were obtained. A second step could be performed with the controlled growth of SiO_2_, which built up until completely embedding the iron oxide particle.

### 4.3. Pickering Emulsions

Pickering emulsions, named after Spencer Pickering [65], represent an important method in forming hybrid particles without an extensive use of surfactant molecules. By controlling the thermodynamic properties of the inorganic and organic phases, it is possible to stabilize droplets and particles by means of inorganic particles placed at the interface [66]. Schrade *et al*. [66] have reviewed this specific topic in a recent publication, so we will only highlight here a few representative and interesting examples.

Silica nanoparticles have been used very often in Pickering systems, for instance in the polymerization of poly(methyl methacrylate). Silica particles have been shown to control the final size of the polymer particles [67], similarly to the control of size exerted by surfactants in emulsion systems. Polystyrene hybrid particles with high titania content were prepared by Pickering emulsion polymerization [68]. A titania hydrosol modified by an anionic monomer, sodium styrene sulfonate, was used as both a Pickering agent and photocatalyst. Polystyrene/titania hybrid particles with well-defined core–shell structure were obtained by photocatalytic polymerization. The resulting hybrid particles had a titania content of up to about 20 wt%.

Multiple phase separation events can lead to interesting final structures in hybrid particles. Poly(*N*-isopropylacrylamide)/poly(methyl methacrylate)/silica hybrid capsules were prepared by inverse Pickering emulsion polymerization. The capsule wall contained two layers—a solid particle monolayer and a polymer layer—and the wall thickness could be controlled by adjusting the methyl methacrylate concentrations in the continuous oil phase. Temperature-responsive properties were observed, which are suitable for controlled release [69].

By using surface modification as a way to control the location of inorganic particles, the same inorganic particles can be placed both within the core polymer matrix and at the surface. A one step synthesis of Fe_3_O_4_-based hybrid materials with Fe_3_O_4_ present *at, on,* and *within* the polymer particle could be produced by modifying the inorganic particles with a functionalizing agent [70]. Fe_3_O_4_ nanoparticles modified with cetyltrimethylammonium bromide acted as a Pickering agent, while Fe_3_O_4_ coated with oleic acid stayed primarily within the monomer droplets. Styrene monomer was then polymerized into polystyrene particles, freezing the oleic acid magnetite in place. The inner particles gave magnetic separability to the hybrid materials, while the interfacial inorganic particles gave catalytic properties. Pickering emulsions with two types of quantum dots were stabilized by silica nanoparticles labeled with fluorescein. Quantum dots were encapsulated during the polymerization process. The hybrid particles showed dual excitation properties [71].

An intriguing subsection of Pickering emulsions is the use of Janus nanoparticles as the Pickering agent. During his Nobel Prize talk, Pierre-Gilles de Gennes [72] coined the term “Janus grains” to describe asymmetric particles, giving rise to what has come to be called “Janus particles”. Janus particles may be composed of polymers and inorganic particles of different elements, and may display two-fold isotropic properties of any desire composition, polarity, color, *etc*. Interesting morphologies can occur, including spherical, cylindrical, disc-like, snowman-, hamburger-, and raspberry-like structures.

Janus nanoparticles can be used as stabilizers in emulsion polymerization along with a surfactant, present on the surface of the inorganic particle, to enhance stabilization of the Pickering emulsion. The Pickering effect was combined with amphiphilicity by using the Janus character of hybrid particles [73]. The adsorption energy at the interface is expected to be significantly higher than that for standard particles, so that Janus particles may suppress undesired aggregation and coalescence more efficiently and for longer times. The increased stabilizing effect, proved through experiments by using Janus particles as a Pickering agent, suggests the ease in which this system may be carried over to hybrid particles.

## 5. *In Situ* Formation of the Inorganic Component in the Presence of Preformed Polymers and Polymer Particles

In the works presented so far, the inorganic component has been always formed *ex situ* and then combined, in one way or another, with the polymer or polymer particles. Strategies following this principle have the advantage of controlling the size and morphology of the inorganic nanoparticles. However, different reaction steps are required, often involving a change of solvent. The re-suspension of the inorganic nanoparticles after preparation in the solvent in which the formation of the hybrid nanoparticle occurs may not be trivial, and the aggregation and segregation become problems to overcome. Therefore, the *in situ* formation of the inorganic component may be in many cases an alternative to be taken into account, not only in terms of practicality but, more importantly, because it provides morphologies and size scales that would not be available by any other strategy.

The formation of inorganic materials in the presence of polymer and polymer particles, can mainly be distinguished into two possibilities: (i) those cases in which polymers act as structuring agents or “soft templates” for the inorganic precipitation/crystallization; and (ii) the formation of the inorganic component on the surface of polymer particles, either by deposition on “bare” particles without any functionalization or on particles functionalized with oligomeric or polymeric chains.

### 5.1. Polymers as Structuring Agents and “Soft Templates”

In the simplest situation, hydrophilic polymers in solution can act as additive and controlling agents in the precipitation of inorganic materials from aqueous media [74–77], so that they become incorporated in an inorganic matrix during the growth, which results in a polymer/inorganic hybrid. This case is commonly referred as “polymer-assisted” or “polymer-controlled crystallization” [78–80]. Sometimes the polymer chains can also act as structuring agents and orient the attachment of the formed mineral nanoparticles to bigger and highly ordered structures, forming so-called “mesocrystals” [81,82]. Biominerals in nature tend to be very good examples of mesocrystals. Nevertheless, the hybrid materials resulting from strategies of polymer-controlled crystallization are typically in the micrometric scale and, thus, beyond the scope of this article.

In a more “sophisticated” situation, polymers are able to assemble in solution (homophase) and in heterophase systems to form a variety of structures: micelles, micellar aggregates, vesicles, stabilized droplets, *etc*. These confined geometries can be used as scaffolds or soft templates for the precipitation and crystallization of inorganic matter and, in general, become occluded into the structure during the formation of the inorganic material. The crystalline properties of both inorganic and organic materials under confinement have been shown to be different from those in the bulk [83,84].

The use of colloidal polymer templates has been recently reviewed by our group (Section 4 in [22]). Here, we will only briefly indicate the three possible cases involving the confinement of sol–gel processes and crystallization to the regions delimited by the templating polymers:

(1) *Confinement in micelles, vesicles, and complex micelles*. By using micellar structures formed by polymers, different research groups have reported the formation of hollow particles of different materials, such as Cu_2_O in the presence of poly(ethylene glycol) [85] or M_1−_*_x_*Fe_2+_*_x_*O_4_ (M = Fe, Co, Mn) in the presence of Pluronic polymers (poly(ethylene oxide)-*block*-poly(propylene oxide)-*block*-poly(ethylene oxide) triblock copolymers) [86]. The use of polymer vesicles in inorganic particle formation has been recently review by Hao *et al. [17].*
(2) *Confinement in emulsion droplets*. The use of emulsion droplets, stabilized by the presence of surfactants—sometimes amphiphilic copolymers—is certainly not the most common way to prepare hybrid particles, but rather to prepare inorganic nanoparticles [18]. The presence of remaining surfactant in the final materials is often an undesired feature that may limit the properties of the inorganic component. However, it is a fact that the inorganic nanoparticles prepared by emulsion methods are not truly inorganic, but “organic–inorganic hybrids”. Far from being always a drawback, in some cases the hybrid character may be advantageous, for example in applications in which hydrophobic particles dispersed in an organic medium are required. The presence of an incorporated polymer may contribute positively to the dispersability and the colloidal stability.
(3) *Microgel particles as soft scaffolds*. Antonietti *et al*. [87] were probably the first to show the application of microgel particles as nanoreactors for the *in situ* synthesis of noble metal nanoparticles, which lead to the formation of hybrid materials. Kumacheva’s research group also used microgel particles in the preparation of metal nanoparticles and CdS [88]. The field has expanded in recent years and a variety of hybrid particles of different compositions have been formed [89–92].

### 5.2. Inorganic Precipitation on the Surface of Polymer Particles

Almost a quarter of a century ago, the group of Egon Matijević published two seminal works on the deposition of *in situ* formed Y(OH)CO_3_ [93] and zirconium compounds (Zr_2_O_2_(OH)_2_CO_3_, Zr_2_(OH)_6_SO_4_) [94] on the surface of cationic and anionic polystyrene latex colloids in the submicrometric scale. The authors explained the process through a heterocoagulation of the forming inorganic nanoparticles (see Figure 5 in Section 3.2.1) to the polymer surface and a subsequent growth, so that the resulting hybrid particles presented a core–shell morphology. A few years later, Tamai and Yasuda [95] reported the formation of hydroxyapatite on the surface of similar polystyrene latexes. In their case, however, the styrene was copolymerized with acrylic acid, resulting in a functionalization of the particle surface with carboxylic groups, which are able to bind to Ca^2+^ ions. The formation of hybrid particles by precipitation of an inorganic component on the surface of latex particles has been a very recurrent strategy, and the advances since these initial works have been considerable.

Using the negative charges from the surfactant used in the latex preparation, different research groups have reported the formation of metallic nanoparticles (such as Ag or Au) [96] and metal oxides [97]. A more common approach, however, is to functionalize the latex surface with polyelectrolytes. This functionalization can be either noncovalent, using the layer-by-layer (LbL) technique, or covalent, by a copolymerization with a functional monomer or by grafting of polyeletrolytic chains. A representative list of the most significant publications in recent years dealing with the formation of inorganic materials on the surface of polymer particles is given in Table 2. It has to be noted that very often the deposition of inorganic materials on polymer spheres is not used to prepare hybrid particles but rather to obtain inorganic hollow structures, after removal of the polymer core by dissolution or calcination. Nevertheless, initially the particles are “hybrid” and could be also used as such.

The LbL technique, already discussed in Section 3.1.1, has been typically used for relatively large microspheres but a few examples of *in situ* precipitation of inorganic materials on the surface of submicrometer particles has also been reported. Oppositely charged polyelectrolytes, such as poly(styrene sulfonate) and poly(alkylammonium chloride), are sequentially deposited on polymer particles and afterwards metal salts are loaded. The precipitation/crystallization process takes places by addition of a precipitating agent (e.g., a reducing agent or a base). By this method, different inorganic materials have been precipitated on the surface of polymer (mostly polystyrene) particles, including gold [101], TiO_2_ [116], and LibNbO_3_ [112].

Unfortunately, the addition of precipitating agents required for an *in situ* formation reaction may in some cases destabilize the polyelectrolyte layers formed by the LbL method. This could be—at least in part—avoided by covalent attachment of the polyelectrolytes to the core polymer sphere. In addition, the use of covalent-functionalization pathways allows for the preparation of typically smaller particles, from a few hundreds of nanometers up to less than 100 nm in the lower range. Once more, a significant part of the work conducted with covalently functionalized nanoparticles involves noble metal particles, obtained by reducing metal salts.

In the origins of such experiments, taking into account that polymer brushes may not be easily observed by electron microscopy, the *in situ* precipitation of tiny noble metal particles on the surface of “hairy” latex particles was used as a method to prove that the attachment of the “hairs” (*i.e.*, the polyelectrolytic chains) had been successful. However, it was rapidly realized that the resulting polymer/inorganic hybrid materials could be also very useful for practical applications, such as catalysis. In this context, Ballauff’s research group has been one of the most successful and prolific in the production of polymer/metal hybrid particles [98,99,103,113,114]. Combining the use of microgels as scaffolds mentioned before, microgel brushes have also been attached to polystyrene particles and used for the precipitation of noble metals [102,124].

Another possibility for covalent functionalization of polymer particles is the copolymerization with a functional comonomer (e.g., acrylic acid copolymerized with styrene). Nanoparticles prepared by this way have been used as supports for the precipitation of calcium phosphates, several metal oxides, and metal sulfides (see references in Table 2).

In general, emulsion and miniemulsion methods used to prepare functional polymer particles involve the use of surfactants, which may affect the binding of metal ions and, thus, the precipitation process. A very elegant way to avoid the effect of additional surfactants has been the use of so-called surfmers (*surf*actant + mono*mer*), surface active monomers able to act simultaneously as a surfactant and as a functionalizing monomer. The use of phosphonate and phosphate surfmers has been recently reported to be a successful strategy to obtain particles that are afterwards used as supports for the controlled precipitation of hydroxyapatite [107] and different metal oxides (CeO_2_, *α*-Fe_2_O_3_, Fe_3_O_4_, ZnO) [109].

The preparation of multifunctional materials is possible by combination of *in situ* crystallization on the surface with the encapsulation of the inorganic components in the polymer core, described in Section 4.1. By using this method, Fischer *et al*. [108] reported in a recent work the synthesis of bifunctional nanoparticles containing an optically active material (CdS) on the surface and a magnetic functionality resulting from the encapsulated magnetite. The preparation of such hybrid multifunctional particles is schematically represented in Figure 9. In this work, cadmium sulfide/Fe_3_O_4_/polymer multifunctional hybrid nanoparticles were prepared by crystallizing cadmium sulfide in a controlled manner on the surface of phosphonate-functionalized polystyrene particles, which contained a magnetic core. A pre-emulsion containing styrene and a phosphonate-functionalized surface-active monomer were mixed with a second pre-emulsion containing magnetite nanoparticles capped with oleic acid. Cadmium sulfide was precipitated from the phosphonate groups on the surface of the polymer particles. The resulting hybrid particles show a “raspberry-like” structure, with superparamagnetic behavior and quantum dot fluorescence.

## 6. Strategies for the Formation of Hybrid Nanoparticles by Simultaneous Polymerization and Inorganic Nanoparticle Precipitation

In Section 4 and Section 5, we have presented strategies in which either the polymer or the inorganic component of the hybrid nanoparticle is formed *in situ*. In principle, the simultaneous formation of both components would also be imaginable. However, this “one-pot” preparation is complex, because the conditions for polymerization and precipitation of inorganic materials are moslty very different. The examples of “all *in situ*” strategies are indeed almost nonexistent so far.

One of the few cases was presented by Fukui and Fujimoto [125], who reported the formation of calcium carbonate in parallel to the polymerization of 2-hydroxyethyl methacrylate (HEMA) in water-in-oil systems. It is know that CaCO_3_ presents different thermodynamically unstable polymorphs that can be stabilized by addition of additives [126]. In their work, Fukui and Fujimoto [125] monitored the formation of these unstable crystals and their transition over time (and with annealing) to more stable polymorphs by “freezing” the evolution of CaCO_3_. CaCO_3_ was formed from precursor droplets containing Ca(NO_3_)_2_ and Na_2_CO_3_, which were first separately formed into droplets by emulsification in the presence of HEMA, followed by mixing of the two pre-emulsions by a final emulsion. The evolution of CaCO_3_ crystals was observed by initiating the polymerization of HEMA via addition of an initiator after a certain amount of time, as seen in Figure 10. Interesting rod-like morphologies and the formation of amorphous calcium carbonate could be achieved [125].

In another example, hybrid capsules containing precipitated metal salts were formed *in situ* by the addition of toluene diisocyanate and a precipitating agent. The resulting poly(urethane–urea) capsule structures trapped the inorganic crystals in the aqueous disperse phase [127].

## 7. Assembly of Hybrid Nanoparticles

The assembly of nanoparticles into organized structures is important in the design of next generation materials that can form hierarchical structures. In the examples of Section 3.2, assemblies of nanoparticles of different natures had been shown to lead to hybrid structures. The arrangement of nanoparticles can also be used as a preparation strategy to obtain hybrid nanoparticles. In this sense, Yu *et al*. [128] described a versatile strategy for engineering binary and ternary hybrid particles through a combination of *etc*hing and deposition processes based on colloidal lithography (Figure 11). Utilizing chemical or plasmonic *etc*hing procedures, polystyrene particles were attached to a gold layer, *etc*hed to reduce their diameter in a controlled way and gold patches were generated underneath the colloidal template, where the attachment on the microspheres was successfully optimized by thermal treatment. The hybrid particles composing metals and polymers were tunable in size, composition, and morphology.

However, hybrid nanoparticles on their own can also be assembled into complex structures. Self-assembly is often a main benefit of adding a polymeric layer around inorganic nanoparticles and has been shown in polymeric colloids for years. In addition, self-assembly of hybrid particles can be taken advantage of to attain unique arrays in which the properties of the inorganic material may be utilized either while still coated, or after removal of the organic phase [129]. Decoration of nanoparticles with linker molecules and polymers is crucial to the formation of complex self-assembled structures, and linkers such as DNA have been shown to form highly controllable structures due to one-to-one matching selectivity in forming a double stranded helix [130]. Kumacheva’s research group has made a significant contribution in understanding how controlled assembly of colloids takes place and finds parallels with macromolecular chemistry [131].

Recently, Bannwarth *et al*. [132] had prepared magnetite particles coated with polystyrene by using the miniemulsion technique. These particles were then passed through a magnet in a temperature controlled pump continuously until chains were acquired. By changing the temperature of the bath, the particles could be fused together to form permanent chains that are entangled with one another or completely fused into fibers. Depending on the content of iron oxide the type of 1D assembly could be tuned to be alternating “zigzag” like patterns to linear fibers.

Clearly, there are many different ways for organizing hybrid particles. The “holy grail” would be a selective organization by having “patchy particles” organize themselves into 3D hierarchical structures based on easily tunable parameters such as temperature [133,134].

## 8. Conclusion and Outlook

Polymer/inorganic hybrid nanoparticles are found in many technological fields, ranging from coatings and catalysis to highly demanding applications, such as optoelectronics and biomedicine. Hybrid nanoparticles are able to combine the features of the integrated components to provide novel materials with enhanced and unique properties. In this review, we have described and exemplified four different synthetic routes for the preparation of polymer/inorganic particles on the nanometric scale, basing our classification on whether the components are formed *in situ* or *ex situ* and on whether the attachment takes place by covalent bonding or by physical means.

Noncovalent approaches, such as heterocoagulation or layer-by-layer deposition are very well-established and have been shown to be very successful in the preparation of highly controlled morphologies. However, the conditions required for certain applications may lead to a destabilization of noncovalent attachments. The covalent binding of polymers and inorganic nanoparticles is a convenient alternative. A lot of effort has been paid to the coupling of polymer chains to inorganic nanoparticles, especially in the context of the so-called bioconjugation for biomedical applications. On the contrary, very few works have been reported on the hierarchical attachment of nanoparticles of different natures. This is certainly a field to be further explored in the upcoming years and opens very interesting possibilities for the preparation of novel materials by assembly of functionalized nanoparticles.

In the case of polymerization in the presence of surface-modified inorganic nanoparticles, one of the key aspects still not understood is the relationship between the functionalization of the particles and the final structure of the hybrids. A better understanding will help in obtaining novel structures, such as Janus and patchy particles, which have become a trend in recent years.

Methods involving *in situ* precipitation have the simplicity and the range of size possibilities as big advantages, but the control of the final morphologies is hard and remains a challenge. In addition, the preparation of such systems takes place under relatively mild conditions, which limits significantly the chemical compositions and inorganic phases that can be achieved. Further research should be undertaken to investigate methods under “non-mild” conditions (higher temperature and pressure).

The preparation of hybrid nanoparticles by “all *in situ*” methods (*i.e.*, by simultaneous polymerization and formation of the inorganic component) remains an unexplored field to be taken into consideration, not only in terms of the convenience of “one-pot” synthesis, but also because it should allow an interpenetration of the polymeric and inorganic component at a size scale that is not possible to be obtained by other methods.

## References

[1] IUPAC (1997). Compendium of Chemical Terminology: Gold Book.

[2] Caruso F. (2001). Nanoengineering of particle surfaces. Adv. Mater..

[3] Caruso F. (2004). Colloids and Colloid Assemblies: Synthesis, Modification, Organization and Utilization of Colloid Particles.

[4] Shi X.Y., Shen M.W., Möhwald H. (2004). Polyelectrolyte multilayer nanoreactors toward the synthesis of diverse nanostructured materials. Prog. Polym. Sci..

[5] Ballauff M., Lu Y. (2007). “Smart” nanoparticles: Preparation, characterization and applications. Polymer.

[6] Lattuada M., Hatton T.A. (2007). Preparation and controlled self-assembly of Janus magnetic nanoparticles. J. Am. Chem. Soc..

[7] Karg M., Hellweg T. (2009). New “smart” poly(NIPAM) microgels and nanoparticle microgel hybrids: Properties and advances in characterisation. Curr. Opin. Colloid Interface Sci..

[8] Landfester K. (2009). Miniemulsion polymerization and the structure of polymer and hybrid nanoparticles. Angew. Chem. Int. Edit. Engl..

[9] Agrawal M., Gupta S., Stamm M. (2011). Recent developments in fabrication and applications of colloid based composite particles. J. Mater. Chem..

[10] Landfester K., Weiss C.K. (2010). Encapsulation by miniemulsion polymerization. Adv. Polym. Sci..

[11] Sperling R.A., Parak W.J. (2010). Surface modification, functionalization and bioconjugation of colloidal inorganic nanoparticles. Philos. Trans. R. Soc. A Math. Phys. Eng. Sci..

[12] Thanh N.T.K., Green L.A.W. (2010). Functionalisation of nanoparticles for biomedical applications. Nano Today.

[13] Hu J., Chen M., Wu L.M. (2011). Organic-inorganic nanocomposites synthesized via miniemulsion polymerization. Polym. Chem..

[14] Musyanovych A., Landfester K. (2007). Core-shell particles. Macromolecular Engineering: Precise Synthesis, Materials Properties, Applications.

[15] Neoh K.G., Kang E.T. (2011). Functionalization of inorganic nanoparticles with polymers for stealth biomedical applications. Polym. Chem..

[16] Chapel J.P., Berret J.F. (2012). Versatile electrostatic assembly of nanoparticles and polyelectrolytes: Coating, clustering and layer-by-layer processes. Curr. Opin. Colloid Interface Sci..

[17] Dong R.H., Liu W.M., Hao J.C. (2012). Soft vesicles in the synthesis of hard materials. Acc. Chem. Res..

[18] Muñoz-Espí R., Weiss C.K., Landfester K. (2012). Inorganic nanoparticles prepared in miniemulsion. Curr. Opin. Colloid Interface Sci..

[19] Sailor M.J., Park J.-H. (2012). Hybrid nanoparticles for detection and treatment of cancer. Adv. Mater..

[20] Froimowicz P., Muñoz-Espí R., Landfester K., Musyanovych A., Crespy D. (2013). Surface-functionalized particles: From their design and synthesis to materials science and bio-applications. Curr. Org. Chem..

[21] He J., Liu Y., Hood T.C., Zhang P., Gong J., Nie Z. (2013). Asymmetric organic/metal(oxide) hybrid nanoparticles: Synthesis and applications. Nanoscale.

[22] Muñoz-Espí R., Mastai Y., Gross S., Landfester K. (2013). Colloidal systems for crystallization processes from liquid phase. CrystEngComm.

[23] Rangelov S., Pispas S. (2014). Polymer and Polymer-Hybrid Nanoparticles: From Synthesis to Biomedical Applications.

[24] Sukhorukov G.B., Donath E., Lichtenfeld H., Knippel E., Knippel M., Budde A., Möhwald H. (1998). Layer-by-layer self assembly of polyelectrolytes on colloidal particles. Colloid Surf. A.

[25] Clark S.L., Hammond P.T. (1998). Engineering the microfabrication of layer-by-layer thin films. Adv. Mater..

[26] Tong W., Song X., Gao C. (2012). Layer-by-layer assembly of microcapsules and their biomedical applications. Chem. Soc. Rev..

[27] Wu P., Zhang H., Du N., Ruan L., Yang D. (2009). A versatile approach for the synthesis of ZnO nanorod-based hybrid nanomaterials via layer-by-layer assembly. J. Phys. Chem. C.

[28] Caruso F., Caruso R.A., Möhwald H. (1998). Nanoengineering of inorganic and hybrid hollow spheres by colloidal templating. Science.

[29] Nakamura M., Katagiri K., Koumoto K. (2010). Preparation of hybrid hollow capsules formed with Fe_3_O_4_ and polyelectrolytes via the layer-by-layer assembly and the aqueous solution process. J. Colloid Interface Sci..

[30] Edmondson S., Osborne V.L., Huck W.T.S. (2004). Polymer brushes via surface-initiated polymerizations. Chem. Soc. Rev..

[31] MILNER S.T. (1991). Polymer brushes. Science.

[32] Zhao B., Brittain W.J. (2000). Polymer brushes: Surface-immobilized macromolecules. Prog. Polym. Sci..

[33] Huang H., Rankin S.E., Penn L.S., Quirk R.P., Cheong T.H. (2004). Transition from mushroom to brush during formation of a tethered layer. Langmuir.

[34] Hui C.M., Pietrasik J., Schmitt M., Mahoney C., Choi J., Bockstaller M.R., Matyjaszewski K. (2014). Surface-initiated polymerization as an enabling tool for multifunctional (nano-)engineered hybrid materials. Chem. Mater..

[35] Pyun J., Kowalewski T., Matyjaszewski K. (2003). Synthesis of polymer brushes using atom transfer radical polymerization. Macromol. Rapid Commun..

[36] Pyun J., Matyjaszewski K. (2001). Synthesis of nanocomposite organic/inorganic hybrid materials using controlled/“living” radical polymerization. Chem. Mater..

[37] Erathodiyil N., Ying J.Y. (2011). Functionalization of inorganic nanoparticles for bioimaging applications. Acc. Chem. Res..

[38] Kolb H.C., Finn M.G., Sharpless K.B. (2001). Click chemistry: Diverse chemical function from a few good reactions. Angew. Chem. Int. Edit. Engl..

[39] Tchoul M.N., Fillery S.P., Koerner H., Drummy L.F., Oyerokun F.T., Mirau P.A., Durstock M.F., Vaia R.A. (2010). Assemblies of titanium dioxide-polystyrene hybrid nanoparticles for dielectric applications. Chem. Mater..

[40] Rosilo H., Kontturi E., Seitsonen J., Kolehmainen E., Ikkala O. (2013). Transition to reinforced state by percolating domains of intercalated brush-modified cellulose nanocrystals and poly(butadiene) in cross-linked composites based on thiol–ene click chemistry. Biomacromolecules.

[41] Fu R., Fu G.-D. (2011). Polymeric nanomaterials from combined click chemistry and controlled radical polymerization. Polym. Chem..

[42] Wagner C.S., Shehata S., Henzler K., Yuan J., Wittemann A. (2011). Towards nanoscale composite particles of dual complexity. J. Colloid Interface Sci..

[43] Kanahara M., Shimomura M., Yabu H. (2014). Fabrication of gold nanoparticle-polymer composite particles with raspberry, core-shell and amorphous morphologies at room temperature via electrostatic interactions and diffusion. Soft Matter.

[44] Jiang S., Chen Q., Tripathy M., Luijten E., Schweizer K.S., Granick S. (2010). Janus particle synthesis and assembly. Adv. Mater..

[45] Agrawal M., Rubio-Retama J., Zafeiropoulos N.E., Gaponik N., Gupta S., Cimrova V., Lesnyak V., López-Cabarcos E., Tzavalas S., Rojas-Reyna R. (2008). Switchable photoluminescence of CdTe nanocrystals by temperature-responsive microgels. Langmuir.

[46] Krovi S.A., Smith D., Nguyen S.T. (2010). “Clickable” polymer nanoparticles: A modular scaffold for surface functionalization. Chem. Commun..

[47] Jang J., Kim J.-K., Choi J.-W., Hwang T.-S., Jo M., Kim I., Cho B.-K., Lee E. (2013). Organic–inorganic vesicular hybrids driven by assembly of dendritic amphiphiles: Site-selective encapsulation of nanoparticles. Chem. Commun..

[48] Luo Q., Hickey R.J., Park S.-J. (2013). Controlling the location of nanoparticles in colloidal assemblies of amphiphilic polymers by tuning nanoparticle surface chemistry. ACS Macro Lett..

[49] Li W., Liu S., Deng R., Wang J., Nie Z., Zhu J. (2013). A simple route to improve inorganic nanoparticles loading efficiency in block copolymer micelles. Macromolecules.

[50] Ischakov R., Adler-Abramovich L., Buzhansky L., Shekhter T., Gazit E. (2013). Peptide-based hydrogel nanoparticles as effective drug delivery agents. Biorg. Med. Chem..

[51] Bockstaller M.R., Mickiewicz R.A., Thomas E.L. (2005). Block Copolymer nanocomposites: Perspectives for tailored functional materials. Adv. Mater..

[52] Hood M.A., Gold C.S., Beyer F.L., Sands J.M., Li C.Y. (2013). Extraordinarily high plastic deformation in polyurethane/silica nanoparticle nanocomposites with low filler concentrations. Polymer.

[53] Romio A.P., Rodrigues H.H., Peres A., Da Cas Viegas A., Kobitskaya E., Ziener U., Landfester K., Sayer C., Araújo P.H.H. (2013). Encapsulation of magnetic nickel nanoparticles via inverse miniemulsion polymerization. J. Appl. Polym. Sci..

[54] Hecht L.L., Schoth A., Muñoz-Espí R., Javadi A., Köhler K., Miller R., Landfester K., Schuchmann H.P. (2013). Determination of the ideal surfactant concentration in miniemulsion polymerization. Macromol. Chem. Phys..

[55] Hecht L.L., Wagner C., Özcan Ö., Eisenbart F., Köhler K., Landfester K., Schuchmann H.P. (2012). Influence of the surfactant concentration on miniemulsion polymerization for the preparation of hybrid nanoparticles. Macromol. Chem. Phys..

[56] Bourgeat-Lami E., Farzi G.A., David L., Putaux J.L., McKenna T.F.L. (2012). Silica encapsulation by miniemulsion polymerization: Distribution and localization of the silica particles in droplets and latex particles. Langmuir.

[57] Hecht L.L., Merkel T., Schoth A., Wagner C., Köhler K., Muñoz-Espí R., Landfester K., Schuchmann H.P. (2013). Emulsification of particle loaded droplets with regard to miniemulsion polymerization. Chem. Eng. J..

[58] Ramirez L.P., Landfester K. (2003). Magnetic polystyrene nanoparticles with a high magnetite content obtained by miniemulsion processes. Macromol. Chem. Phys..

[59] Urban M., Musyanovych A., Landfester K. (2009). Fluorescent Superparamagnetic polylactide nanoparticles by combination of miniemulsion and emulsion/solvent evaporation techniques. Macromol. Chem. Phys..

[60] Perro A., Reculusa S., Bourgeat-Lami E., Duguet E., Ravaine S. (2006). Synthesis of hybrid colloidal particles: From snowman-like to raspberry-like morphologies. Colloids Surf. A Physicochem. Eng. Asp..

[61] Reculusa S., Mingotaud C., Bourgeat-Lami E., Duguet E., Ravaine S. (2004). Synthesis of daisy-shaped and multipod-like silica/polystyrene nanocomposites. Nano Lett..

[62] Negrete-Herrera N., Putaux J.-L., David L., De Haas F., Bourgeat-Lami E. (2007). Polymer/Laponite composite latexes: Particle morphology, film microstructure, and properties. Macromol. Rapid Commun..

[63] Qiang W., Wang Y., He P., Xu H., Gu H., Shi D. (2008). Synthesis of asymmetric inorganic/polymer nanocomposite particles via localized substrate surface modification and miniemulsion polymerization. Langmuir.

[64] Feyen M., Weidenthaler C., Schueth F., Lu A.-H. (2010). Regioselectively controlled synthesis of colloidal mushroom nanostructures and their hollow derivatives. J. Am. Chem. Soc..

[65] Pickering S.U. (1907). Emulsions. J. Chem. Soc. Trans..

[66] Schrade A., Landfester K., Ziener U. (2013). Pickering-type stabilized nanoparticles by heterophase polymerization. Chem. Soc. Rev..

[67] Pakdel A., Pourmahdian S., Eslami H. (2012). One-pot preparation of core–shell, organic–inorganic, hybrid latexes by *in situ* nanoparticle precipitation in pickering emulsion polymerization. Macromol. Chem. Phys..

[68] Song X., Yin G., Zhao Y., Wang H., Du Q. (2009). Effect of an anionic monomer on the pickering emulsion polymerization stabilized by titania hydrosol. J. Polym. Sci. A Polym. Chem..

[69] Zhang K., Wu W., Guo K., Chen J., Zhang P. (2010). Synthesis of temperature-responsive poly(*N*-isopropyl acrylamide)/poly(methyl methacrylate)/silica hybrid capsules from inverse pickering emulsion polymerization and their application in controlled drug release. Langmuir.

[70] Yin D., Du X., Liu H., Zhang Q., Ma L. (2012). Facile one-step fabrication of polymer microspheres with high magnetism and armored inorganic particles by Pickering emulsion polymerization. Colloid Surf. A.

[71] Kang B., Schrade A., Xu Y., Chan Y., Ziener U. (2012). Synthesis and characterization of dually labeled pickering-type stabilized polymer nanoparticles in a downscaled miniemulsion system. Langmuir.

[72] De Gennes P.-G. (1991). Soft Matter.

[73] Walther A., Hoffmann M., Müller A.H.E. (2008). Emulsion polymerization using janus particles as stabilizers. Angew. Chem. Int. Edit. Engl..

[74] Muñoz-Espí R., Qi Y., Lieberwirth I., Gómez C.M., Wegner G. (2006). Surface-functionalized latex particles as controlling agents for the mineralization of zinc oxide in aqueous medium. Chem. Eur. J..

[75] Muñoz-Espí R., Jeschke G., Lieberwirth I., Gómez C.M., Wegner G. (2007). ZnO-latex hybrids obtained by polymer-controlled crystallization: A spectroscopic investigation. J. Phys. Chem. B.

[76] Cölfen H., Naka K. (2007). Bio-inspired mineralization using hydrophilic polymers. Biomineralization II: Mineralization Using Synthetic Polymers and Templates.

[77] Gorna K., Muñoz-Espí R., Gröhn F., Wegner G. (2007). Bioinspired mineralization of inorganics from aqueous media controlled by synthetic polymers. Macromol. BioSci..

[78] Taubert A., Palms D., Weiss O., Piccini M.T., Batchelder D.N. (2002). Polymer-assisted control of particle morphology and particle size of zinc oxide precipitated from aqueous solution. Chem. Mater..

[79] Schweizer S., Taubert A. (2007). Polymer-controlled, bio-inspired calcium phosphate mineralization from aqueous solution. Macromol. BioSci..

[80] Muñoz-Espí R., Burger C., Krishnan C.V., Chu B. (2008). Polymer-controlled crystallization of molybdenum oxides from peroxomolybdates: Structural diversity and application to catalytic epoxidation. Chem. Mater..

[81] Cölfen H., Antonietti M. (2005). Mesocrystals: Inorganic superstructures made by highly parallel crystallization and controlled alignment. Angew. Chem. Int. Edit. Engl..

[82] Cölfen H., Antonietti M. (2008). Mesocrystals and Nonclassical Crystallization.

[83] Montenegro R., Antonietti M., Mastai Y., Landfester K. (2003). Crystallization in miniemulsion droplets. J. Phys. Chem. B.

[84] Hood M.A., Wang B., Sands J.M., La Scala J.J., Beyer F.L., Li C.Y. (2010). Morphology control of segmented polyurethanes by crystallization of hard and soft segments. Polymer.

[85] Xu Y., Chen D., Jiao X., Xue K. (2007). Nanosized Cu_2_O/PEG400 composite hollow spheres with mesoporous shells. J. Phys. Chem. C.

[86] Li X.-H., Zhang D.-H., Chen J.-S. (2006). Synthesis of amphiphilic superparamagnetic ferrite/block copolymer hollow submicrospheres. J. Am. Chem. Soc..

[87] Antonietti M., Grohn F., Hartmann J., Bronstein L. (1997). Nonclassical shapes of noble-metal colloids by synthesis in microgel nanoreactors. Angew. Chem. Int. Edit. Engl..

[88] Xu S., Zhang J., Paquet C., Lin Y., Kumacheva E. (2003). From hybrid microgels to photonic crystals. Adv. Funct. Mater..

[89] Sugawara A., Yamane S., Akiyoshi K. (2006). Nanogel-templated mineralization: Polymer-calcium phosphate hybrid nanomaterials. Macromol. Rapid Commun..

[90] Schachschal S., Pich A., Adler H.J. (2008). Aqueous microgels for the growth of hydroxyapatite nanocrystals. Langmuir.

[91] Yamane S., Sugawara A., Watanabe A., Akiyoshi K. (2009). Hybrid nanoapatite by polysaccharide nanogel-templated mineralization. J. Bioact. Compat. Polym.

[92] Zhu H.B., Li Y.X., Qiu R.Q., Shi L., Wu W.T., Zhou S.Q. (2012). Responsive fluorescent Bi_2_O_3_@PVA hybrid nanogels for temperature-sensing, dual-modal imaging, and drug delivery. Biomaterials.

[93] Kawahashi N., Matijević E. (1990). Preparation and properties of uniform coated colloidal particles: V. Yttrium basic carbonate on polystyrene latex. J. Colloid Interface Sci..

[94] Kawahashi N., Persson C., Matijević E. (1991). Zirconium compound as coatings on polystyrene latex and as hollow spheres. J. Mater. Chem..

[95] Tamai H., Yasuda H. (1999). Preparation of polymer particles coated with hydroxyapatite. J. Colloid Interface Sci..

[96] Zhang J.H., Liu J.B., Wang S.Z., Zhan P., Wang Z.L., Ming N.B. (2004). Facile methods to coat polystyrene and silica colloids with metal. Adv. Funct. Mater..

[97] Huang Z.B., Tang F.Q., Zhang L. (2005). Morphology control and texture of Fe_3_O_4_ nanoparticle-coated polystyrene microspheres by ethylene glycol in forced hydrolysis reaction. Thin Solid Films.

[98] Lu Y., Mei Y., Drechsler M., Ballauff M. (2006). Thermosensitive core-shell particles as carriers for Ag nanoparticles: Modulating the catalytic activity by a phase transition in networks. Angew. Chem. Int. Edit. Engl..

[99] Sharma G., Ballauff M. (2004). Cationic spherical polyelectrolyte brushes as nanoreactors for the generation of gold particles. Macromol. Rapid Commun..

[100] Schrinner M., Polzer F., Mei Y., Lu Y., Haupt B., Ballauff M., Goldel A., Drechsler M., Preussner J., Glatzel U. (2007). Mechanism of the formation of amorphous gold nanoparticles within spherical polyelectrolyte brushes. Macromol. Chem. Phys..

[101] Tian C.G., Mao B.D., Wang E.B., Kang Z.H., Song Y.L., Wang C.L., Li S.H. (2007). Simple strategy for preparation of core colloids modified with metal nanoparticles. J. Phys. Chem. C.

[102] Lu Y., Yuan J., Polzer F., Drechsler M., Preussner J. (2010). *In situ* growth of catalytic active Au-Pt bimetallic nanorods in thermoresponsive core−shell microgels. ACS Nano.

[103] Schrinner M., Ballauff M., Talmon Y., Kauffmann Y., Thun J., Möller M., Breu J. (2009). Single nanocrystals of platinum prepared by partial dissolution of Au-Pt nanoalloys. Science.

[104] Ethirajan A., Ziener U., Landfester K. (2009). Surface-functionalized polymeric nanoparticles as templates for biomimetic mineralization of hydroxyapatite. Chem. Mater..

[105] Schachschal S., Pich A., Adler H.J. (2007). Growth of hydroxyapatite nanocrystals on polymer particle surface. Colloid Polym. Sci..

[106] Schöller K., Ethirajan A., Zeller A., Landfester K. (2011). Biomimetic route to calcium phosphate coated polymeric nanoparticles: Influence of different functional groups and pH. Macromol. Chem. Phys..

[107] Sauer R., Froimowicz P., Schöller K., Cramer J.M., Ritz S., Mailänder V., Landfester K., Design Synthesis. (2012). miniemulsion polymerization of new phosphonate surfmers and application studies of the resulting nanoparticles as model systems for biomimetic mineralization and cellular uptake. Chem. Eur. J..

[108] Fischer V., Bannwarth M.B., Jakob G., Landfester K., Muñoz-Espí R. (2013). Luminescent and magnetoresponsive multifunctional chalcogenide/polymer hybrid nanoparticles. J. Phys. Chem. C.

[109] Fischer V., Lieberwirth I., Jakob G., Landfester K., Muñoz-Espí R. (2013). Metal oxide/polymer hybrid nanoparticles with versatile functionality prepared by controlled surface crystallization. Adv. Funct. Mater..

[110] Ohnishi M., Kozuka Y., Ye Q.-L., Yoshikawa H., Awaga K., Matsuno R., Kobayashi M., Takahara A., Yokoyama T., Bandow S., Iijima S. (2006). Phase selective preparations and surface modifications of spherical hollow nanomagnets. J. Mater. Chem..

[111] Agrawal M., Pich A., Gupta S., Zafeiropoulos N.E., Formanek P., Jehnichen D., Stamm M. (2010). Tailored growth of In(OH)_3_ shell on functionalized polystyrene beads. Langmuir.

[112] Wang D., Caruso F. (2002). Polyelectrolyte-coated colloid spheres as templates for sol−gel reactions. Chem. Mater..

[113] Mei Y., Lu Y., Polzer F., Ballauff M., Drechsler M. (2007). Catalytic activity of palladium nanoparticles encapsulated in spherical polyelectrolyte brushes and core-shell microgels. Chem. Mater..

[114] Mei Y., Sharma G., Lu Y., Ballauff M., Drechsler M., Irrgang T., Kempe R. (2005). High catalytic activity of platinum nanoparticles immobilized on spherical polyelectrolyte brushes. Langmuir.

[115] Agrawal M., Pich A., Gupta S., Zafeiropoulos N.E., Simon P., Stamm M. (2008). Synthesis of novel tantalum oxide sub-micrometer hollow spheres with tailored shell thickness. Langmuir.

[116] Caruso F., Shi X., Caruso R.A., Susha A. (2001). Hollow titania spheres from layered precursor deposition on sacrificial colloidal core particles. Adv. Mater..

[117] Yang Z., Niu Z., Lu Y., Hu Z., Han C.C. (2003). Templated synthesis of inorganic hollow spheres with a tunable cavity size onto core–shell gel particles. Angew. Chem. Int. Edit. Engl..

[118] Agrawal M., Pich A., Zafeiropoulos N.E., Stamm M. (2008). Fabrication of hollow titania microspheres with tailored shell thickness. Colloid Polym. Sci..

[119] Agrawal M., Gupta S., Pich A., Zafeiropoulos N.E., Rubio-Retama J., Jehnichen D., Stamm M. (2010). Template-assisted fabrication of magnetically responsive hollow titania capsules. Langmuir.

[120] Lu Y., Hoffmann M., Yelamanchili R.S., Terrenoire A., Schrinner M., Drechsler M., Möller M.W., Breu J., Ballauff M. (2009). Well-defined crystalline TiO_2_ nanoparticles generated and immobilized on a colloidal nanoreactor. Macromol. Chem. Phys..

[121] Agrawal M., Pich A., Zafeiropoulos N.E., Gupta S., Pionteck J., Simon F., Stamm M. (2007). Polystyrene-ZnO composite particles with controlled morphology. Chem. Mater..

[122] Agrawal M., Gupta S., Pich A., Zafeiropoulos N.E., Stamm M. (2009). A facile approach to fabrication of ZnO-TiO_2_ hollow spheres. Chem. Mater..

[123] Pich A., Hain J., Prots Y., Adler H.J. (2005). Composite polymeric particles with ZnS shells. Polymer.

[124] Chen C.-W., Chen M.-Q., Serizawa T., Akashi M. (1998). *In-situ* formation of silver nanoparticles on poly(*N*-isopropylacrylamide)-coated polystyrene microspheres. Adv. Mater..

[125] Fukui Y., Fujimoto K. (2012). Bio-inspired nanoreactor based on a miniemulsion system to create organic-inorganic hybrid nanoparticles and nanofilms. J. Mater. Chem..

[126] Hood M.A., Landfester K., Muñoz-Espí R. (2014). The role of residue acidity on the stabilization of vaterite by amino acids and oligopeptides. Cryst. Growth Des.

[127] Hamberger A., Ziener U., Landfester K. (2013). Encapsulation of *in situ* nanoprecipitated inorganic materials in confined geometries into a polymer shell using inverse miniemulsion. Macromol. Chem. Phys..

[128] Yu Y., Ai B., Moehwald H., Zhou Z., Zhang G., Yang B. (2012). Fabrication of binary and ternary hybrid particles based on colloidal lithography. Chem. Mater..

[129] Lohse S.E., Murphy C.J. (2012). Applications of colloidal inorganic nanoparticles: From medicine to energy. J. Am. Chem. Soc..

[130] Simon U. (2013). Nanoparticle self-assembly: Bonding them all. Nat. Mater..

[131] Choueiri R.M., Klinkova A., Thérien-Aubin H., Rubinstein M., Kumacheva E. (2013). Structural transitions in nanoparticle assemblies governed by competing nanoscale forces. J. Am. Chem. Soc..

[132] Bannwarth M.B., Kazer S.W., Ulrich S., Glasser G., Crespy D., Landfester K. (2013). Well-defined nanofibers with tunable morphology from spherical colloidal building blocks. Angew. Chem. Int. Edit. Engl..

[133] Kraft D.J., Ni R., Smallenburg F., Hermes M., Yoon K., Weitz D.A., van Blaaderen A., Groenewold J., Dijkstra M., Kegel W.K. (2012). Surface roughness directed self-assembly of patchy particles into colloidal micelles. Proc. Natl. Acad. Sci. USA.

[134] McConnell M.D., Kraeutler M.J., Yang S., Composto R.J. (2010). Patchy and multiregion janus particles with tunable optical properties. Nano Lett..
